# The Art of Health Visiting

**Published:** 1920-01-03

**Authors:** 


### THE ART OF HEALTH VISITING.

MUCH attention has been recently drawn to the army of workers which is necessary to carry out the far-reaching activities of our public health and school medical services, and this has come not only with the publication of numerous highly important official reports, but also with the appearance of an undoubtedly reactionary element among the poorer people, which tends to protest against the invasion of their homes by a swarm of well-meaning welfare officials whom these reports would have us understand are absolutely essential to the future plans and their success. Recently we drew attention to the very mischievous propaganda which has been set on foot by a body existing under the title of the "Mothers' Defence League," which has for its most apparent object the stirring-up of opposition to the activities of school nurses, health visitors, almoners, and similar helpers of the Ministry. This is but one specific instance. We believe that there are many similar agencies of opposition at work, though at such a transition period as the present, when there is the utmost need for patient pioneer work of explanation and the gradual removal of all such prejudices, any attempt to retard reform is a work for which men and women should be ashamed to combine.

Obviously it is a suicidal policy to allow too great stress to be laid publicly on the people's legal right to exclusion of certain visitors, and the activities of such associations as the "Defence League" should be condemned with the utmost vigour, but let us realise that there is, and would indeed be without the help of any such organised propaganda, a natural and instinctive dislike on the part of many of the working-class population for the entry of visitors of any type to their homes. It is a situation which must be accepted by the authorities and dealt with accordingly. Simply to multiply the number of visitors is a method of increased health supervision doomed from the outset to relative failure. We believe that, if anything, the number of actual "visitors" must be reduced and the apparent deficiency made good by a combination of improved and far more centralised administration, and also by arranging that the "visits" are made by a person of wider experience, better qualifications, and altogether a far more finished and specialised product than is at present often the case. In other words, the Government must more actively interest itself in the type and training of those through whom it intends to minister to the people in the people's homes.

To illustrate our meaning with regard to centralisation, let us notice the greatly improved results obtained in certain localities, particularly in parts of London, where social work, so-called, is carried on through one committee and one only, as in the area scheme which is gaining ever more general approval. How infinitely easier has become that part of general sanitary and health supervision directly controlled by the medical officer of health, the administrative perfection of whose office has generally become annually more definite and perfected. But the need of such organisations, simplification, and the reduction or absorption of a multitude of subsidiary well-meaning associations is too obvious longer to claim our attention. Rather should we consider the types of "visitor" already at large among the poorer classes.

Here we must tread warily. It is far from our intention to offend some of the noblest-hearted, best-intentioned women in the country, but it must be realised that, quite apart from the fact that there is much more both human and highly technical knowledge necessary for efficiency in this work, the art of actually bringing help and useful advice to the lower-class homes, the type of listened-to advice which the Ministry of Health is setting out to promulgate, is not an accomplishment that more than a relatively small proportion of middle-class women—or men either, for that matter—can ever hope to achieve. And one has but to attend an examination of male and female candidates for, say, the sanitary inspectors' examinations, to investigate the working of many most excellent voluntary organisations, to see the process of our present official welfare work in almost any locality, to realise that many of those actively visiting and doing their very utmost to aid the unenlightened poor are of a type foredoomed to failure—failure, at least, to achieve that mutual understanding and sympathy with those they seek to benefit. Education of the people in health matters is essential, but understanding and trust must come first, and our new Ministry or their local representative must take the utmost care in selecting their little army of actual contact.

